# The Unknown Nature of the Antigen in the Direct Agglutination Test for Visceral Leishmaniasis Hampers Development of Serodiagnostic Tests

**DOI:** 10.4269/ajtmh.18-0740

**Published:** 2018-12-17

**Authors:** Vera Kühne, Philippe Büscher

**Affiliations:** Institute of Tropical Medicine, Antwerp, Belgium

## Abstract

Current diagnostic tests for visceral leishmaniasis (VL) are either not adapted for use in resource-poor settings or are insufficiently accurate in Eastern Africa. Only the direct agglutination test (DAT), based on whole *Leishmania* promastigotes, is highly reliable in all endemic regions, but its implementation is hampered by the need for a cold chain, minimal laboratory conditions, and long incubation times. Integrating the DAT antigen(s) in an immunochromatographic rapid diagnostic test (RDT) would overcome these disadvantages. Unfortunately, the identity of the DAT antigen(s) involved in the agglutination reaction is unknown. For this study, we reviewed all publications that might shed some light on this issue. We conclude that the DAT antigen is a mixture of *Leishmania*-specific epitopes of protein, carbohydrate, and lipid nature. To develop an accurate RDT for VL diagnosis in Eastern Africa, we suggest to complement the classical protein antigen discovery with approaches to identify carbohydrate and lipid epitopes.

## INTRODUCTION

With more than 100 years of research on the diagnosis of visceral leishmaniasis (VL), the direct agglutination test (DAT) remains the most reliable serodiagnostic test for the disease in the field.^1^

**Figure 1.**
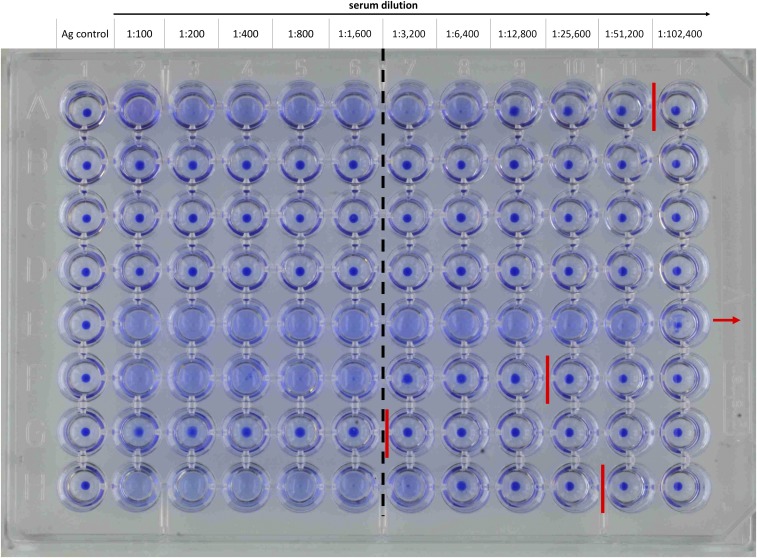
Result of a direct agglutination test (DAT). Serial serum dilutions from 1:100 to 1:102,400 (column two to 12 from left to right) are incubated with DAT antigen overnight. Column 1 is the antigen control without any serum. Different sera are titered in rows A–H. The red lines mark the last dilutions that are considered positive (end-titer) and the red arrow indicates that the titration in the plate was not sufficient to determine the end-titer. The cutoff line (dashed black) is between a dilution of 1:1,600 and 1:3,200. This figure appears in color at www.ajtmh.org.

The DAT as we use it today (with some minor modifications) has been described 30 years ago by El Harith and others,^2^ based on the principle developed by Allain and Kagan 11 years earlier.^3^ The antigen preparation starts with the in vitro culture of *Leishmania* (*L.*) *donovani* LD-1S promastigotes that are harvested during the log phase (noninfectious leptomonads) and subsequently are trypsinized, fixed with formaldehyde, and stained with Coomassie Blue.^4,5^ The final reagent can be provided as aqueous suspension but for improved thermostability, it can be freeze-dried.^4–6^ The test is performed by overnight incubation of DAT antigen with 2-fold serial dilutions of serum (or blood eluted from filter paper) in a β-mercaptoethanol (ME)–containing phosphate-buffered saline, pH 7.2, supplemented with 0.2% serum protein, in a V-bottom microtiter plate. According to Jacquet et al.,^5^ a serum can be considered positive if at a dilution ≥ 1:3,200, agglutinated promastigote cells are visible by naked eye as a blue mat in the microtiter plate wells; absence of agglutination is visible as a neat blue spot in the bottom of the microtiter plate wells (Figure 1). The figure demonstrates the difficulty in assessing the end-titer of a sample, as in rows A, F, G, and H only very close zoom shows the blue dot’s edges to be sharper behind the indicated end-titer. The issue of inter-reader variability has been addressed by Adam et al. (2012)^7^ by introducing pictorials for training purposes. The meta-analysis published by Chappuis et al.^1^ shows that different laboratories apply different cutoff titers, however, without an important effect on the diagnostic performance.

The diagnostic performance of DAT is high in all VL-endemic regions with overall sensitivity and specificity of 95% (93–97%) and 97% (94–99%), respectively, irrespective of the *Leishmania* species causing VL (*L. donovani* or *Leishmania* [*L.*] *infantum*).^1^ However, the large-scale implementation of DAT is hampered by several factors, including the need for well-trained technicians, a cold chain, and a laboratory environment, the long incubation time, and the relatively high price per test. Early problems of reproducibility were also caused by the instability of the DAT antigen as liquid suspension.^8^ Today, liquid DAT antigen has been replaced by freeze-dried antigen, which makes it less sensitive to degradation at elevated temperature.^5^ No large-scale reproducibility studies have been performed with the freeze-dried DAT antigen, but Boelaert and others^9^ found the reproducibility to be good.

Current DAT antigen suppliers, Institute of Tropical Medicine Antwerp and Academic Medical Centre Amsterdam, quote a price ranging from 3.30€ to 8.50€ for testing one serum on 11 dilutions including a negative and positive control per plate. The lyophilized antigen is conditioned for minimum four to eight tests, depending on the supplier.

In parallel to DAT, researchers invested in the development of a rapid diagnostic test (RDT), which would be more compliant with the ASSURED criteria (accurate, sensitive, specific, user friendly, rapid and robust, equipment-free, and delivered to those who need it).^10^ Up to now, the best alternative is the rK39-based immunochromatographic test,^11^ which is highly accurate on the Indian subcontinent (sensitivity and specificity of 97% (90–100%) and 90% (76–98%), respectively) but less sensitive in East Africa (sensitivity and specificity of 85% (75–93%) and 91% (80–97%), respectively).^12^ The rK39 antigen is derived from a genomic library of *L. infantum*.^13^ Cleaved *L. infantum* gDNA was cloned into *Escherichia* (*E.*) *coli* cells to recombinantly express the inserted gene fragments. The clone expressing the rK39 fragment was selected by screening against serum of an *L. donovani*–infected patient. The rK39 fragment consists of 6.4 repeats of a 39-amino acid (aa) stretch belonging to a large kinesin-related protein expressed predominantly by amastigotes.^13^ More recently, an rK28 antigen has been described and incorporated into an RDT format to increase diagnostic accuracy in East Africa. It is a chimeric antigen expressed in *E. coli* composed of three 14-aa repeats of the *L. donovani* hydrophilic acylated surface protein B1 (HASPB1) gene, two 39-aa repeats of the *L. donovani* K39 kinesin protein gene, and the complete open reading frame of *L. donovani* hydrophilic acylated surface protein B2 (HASPB2) gene.^14^ It has so far only been evaluated in three studies on a total of 621 VL patients and 598 controls in Sudan, Ethiopia, and Bangladesh with promising but variable results (sensitivities between 89% and 99% and specificities between 81% and 99%).^14–16^

Another approach to exploit the high accuracy of the DAT antigen would be to incorporate the DAT antigen in an immunochromatographic test format. Unfortunately, the exact nature of the antigen(s) or epitope(s) that lay on the basis of the high diagnostic accuracy of the DAT remains a mystery. Therefore, this study was undertaken to review the existing literature on the DAT antigen.

## SEARCH STRATEGY AND SELECTION CRITERIA

References for this review were identified through searches between January 2017 and July 2017 of PubMed and Google Scholar for articles published until July 2017, by use of the terms “DAT AND *Leishmania*,” “Direct Agglutination Test AND *Leishmania*,” “diagnosis AND *Leishmania*,” “membrane AND *Leishmania*,” “antigen AND *Leishmania*,” “Wasserman test,” “aldehyde test,” “formol-gel test,” “lipophosphoglycan (LPG),” “trypsin AND *Leishmania*,” “beta-mercaptoethanol AND *Leishmania*,” “carbohydrate AND antigen,” and “lipid AND antigen.” Other relevant articles were identified through searches in the authors’ and collaborators’ personal files. Articles resulting from these searches and relevant references cited in those articles were reviewed. Articles published in English, French, and German were included.

## DOES THE DAT TEST DETECT VL-SPECIFIC ANTIBODIES?

Historically, two tests challenge the need for *Leishmania*-specific antigens in the serodiagnosis of VL: the Wasserman test that makes use of bacterial extracts to detect VL with a sensitivity of 74% and a specificity of 75%^17^ and the aldehyde test, or “formol-gel” test, that suggests VL without making use of any particular antigen but that shows surprisingly high diagnostic accuracy; sensitivity of 98% and specificity of 98% in its first description by Napier,^18^ whereas in a more recent study in Nepal by Boelaert et al.,^19^ a sensitivity of 34% (27–41%) and specificity of 99% (96–100%) was detected. By adding formaldehyde to a patient’s serum, an opaque gel develops in the case of VL, whereas for non-VL cases, the serum remains clear and liquid. This solidification reaction with formaldehyde is postulated to be caused by the very high concentration of immunoglobulins in VL patients.^18^

Based on the diagnostic value of these two tests, one can hypothesize that the DAT is just a reinvention of one or a combination of the two tests, as suggested by Hommel et al.^20^ Thus, DAT would detect antibodies against nonspecific epitopes in the antigen preparation (Wasserman test reinvented) and/or detect hypergammaglobulinaemia in VL patients (aldehyde test reinvented) resulting from polyclonal B-cell activation during *Leishmania* infection.^21^ Interestingly, Hommel et al.^20^ were able to mimic the agglutination reaction of the DAT with glutaraldehyde-cross-linked human serum albumin (HSA*Glut) on latex beads. An enzyme-linked immunosorbent assay (ELISA) using HSA*Glut was more specific for the diagnosis of VL than a crude promastigote extract. They further observed agglutination of the DAT antigen by a monoclonal antibody against HSA*Glut. They pointed out that they had no explanation for this as the hypothesis that culture medium–derived albumin sticking to the DAT antigen would be responsible for the DAT reaction is not supported by the observation that promastigotes grown in albumin-free medium still produce agglutination reactions, even with the monoclonal antibody against HSA*Glut.^22^

The fact that DAT antigen contains nonspecific epitopes is illustrated by its reactivity with sera from patients with diseases known to induce high levels of nonspecific antibodies, such as African trypanosomiasis and leukemia.^23^ Lakhal et al. showed that antinuclear antibodies cross-react with *L. infantum*–conserved proteins and Argov et al. could inhibit the binding of autoantibodies to nuclear antigens using (glutaraldehyde) fixed *L. donovani* promastigotes.^24,25^ This suggests a molecular mimicry between *Leishmania* antigens and autoantigens, which would explain binding of non-*Leishmania* antibodies to DAT. Yet, non-VL sera show only marginally positive titers (1:1,600–1:3,200) in DAT, suggesting that there must be high amounts of antibodies in VL-positive sera that react with specific epitopes on the DAT antigen and thus cause agglutination at dilutions above 1:3,200.^23^ Further evidence against the DAT reaction being a reinvention of the Wasserman test comes from the absence of cross-reaction between VL-positive sera and DAT antigen prepared from *Trypanosoma cruzi* epimastigotes, which has been used for serodiagnosis of Chagas disease.^26^

If the DAT detects *Leishmania*-specific antibodies, the question arises which antigens or epitopes are recognized by these antibodies. Because DAT consists of entire promastigote cells, cell surface–exposed antigens are logical antigen candidates.

## THE SURFACE ARCHITECTURE OF *L. DONOVANI* PROMASTIGOTES

**Figure 2.**
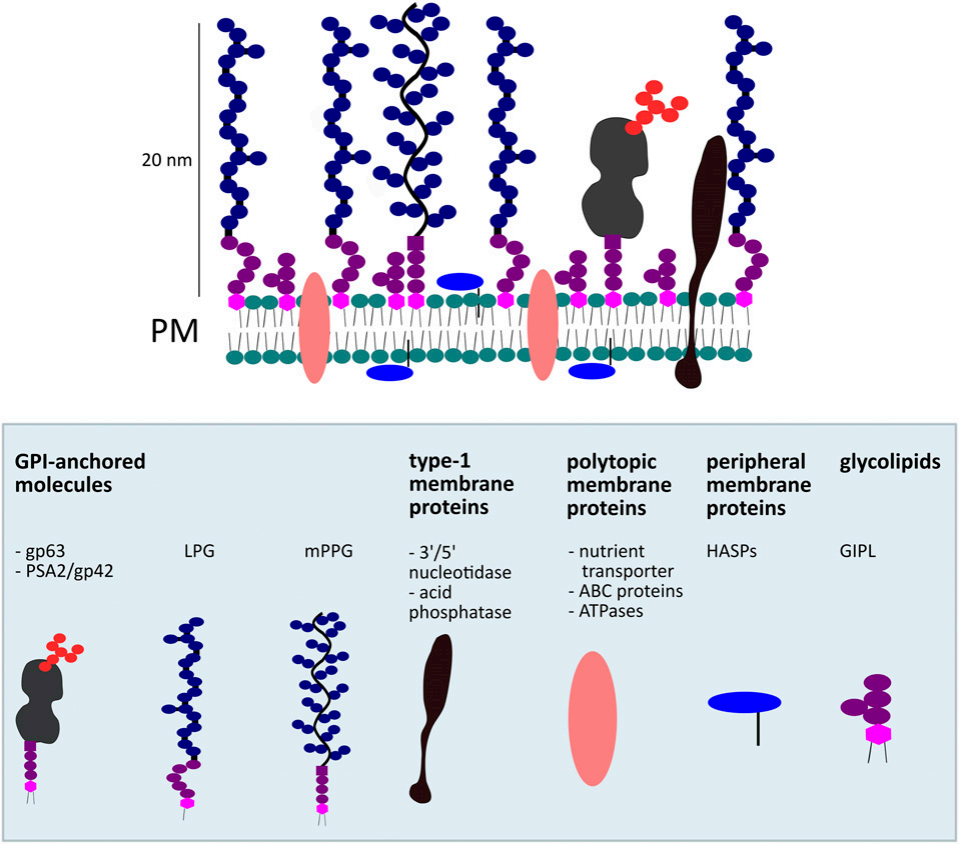
Schematic representation of the promastigote-specific expression of different classes of *Leishmania* plasma membrane components based on Naderer et al.^27^ ATP = adenosine triphosphate; GIPL = glycoinositolphospholipids; GPI = glycosylphosphatidylinositol; HASPB = hydrophilic acylated surface protein B; LPG = lipophosphoglycan; mPPG = proteophosphoglycan; PM = plasma membrane; PSA = promastigote surface antigen. This figure appears in color at www.ajtmh.org.

The surface of a *L. donovani* promastigote cell is covered by a dense glycocalyx of 20–40-nm thickness (Figure 2).^27^ This glycocalyx is composed of LPG, glycosylphosphatidylinositol (GPI)-anchored glycoproteins, and glycoinositolphospholipids. Interestingly, amastigotes lack this prominent glycocalyx.^27^

Lipophosphoglycan is the predominant component of the promastigote glycocalyx, whereas it is absent or undetectable in lesion-derived amastigotes.^27^ It consists of a long and heterogeneous phosphoglycan chain anchored in the membrane by a lipid (reviewed by Turco).^28^ During the development of promastigotes in the sand fly vector from procyclics to infective metacyclics, LPG undergoes a change, metacyclic LPG being longer and incapable of binding to the sand fly midgut as opposed to procyclic LPG.^29^ Glycoinositolphospholipids are also anchored in the membrane by a lipid but possess a much shorter polysaccharide tail.^30^ There are at least three GPI-anchored glycoproteins in the promastigote glycocalyx, namely gp63 (a zinc metalloproteinase), gp46/promastigote surface antigen complex 2, and GPI-anchored proteophosphoglycans, which, like LPG are less abundant in amastigotes. Proteophosphoglycans are a heterogeneous family of cell surface and secrete proteins that are extensively modified with similar phosphoglycan chains to those found in LPG. Apart from the glycocalyx, the surface of a promastigote is composed of a number of membrane proteins: type-1 integral membrane proteins, polytopic membrane proteins (having more than one transmembrane loop), and peripheral membrane proteins (among which HASPB of which two fragments are used as antigens in the rK28 serodiagnostic test).^27^

Given the known composition of the surface of a promastigote cell, there are different hypotheses about the nature of the DAT antigen that come to mind. In the following paragraphs, we will discuss the different possible candidate antigens and compound classes. Most publications on the DAT antigen, however, did not aim to identify the nature of the antigen but rather to improve the test accuracy, which leaves us with an incomplete dataset that mainly allows us to hypothesize on nature of the DAT antigen, rather than to provide conclusive answers.

### Lipophosphoglycan.

As LPG covers the surface of the promastigote, it is a likely antigen candidate, such as lipopolysaccharides in bacteria or variant surface glycoproteins (VSGs) in *Trypanosoma brucei*. Purified LPG in ELISA format indeed has a diagnostic value to detect VL (sensitivity and specificity of 92% tested on 38 Mediterranean VL cases and 108 controls).^31^ Monoclonal antibodies against LPG evoke an agglutination reaction with the DAT antigen.^20^

**Figure 3.**
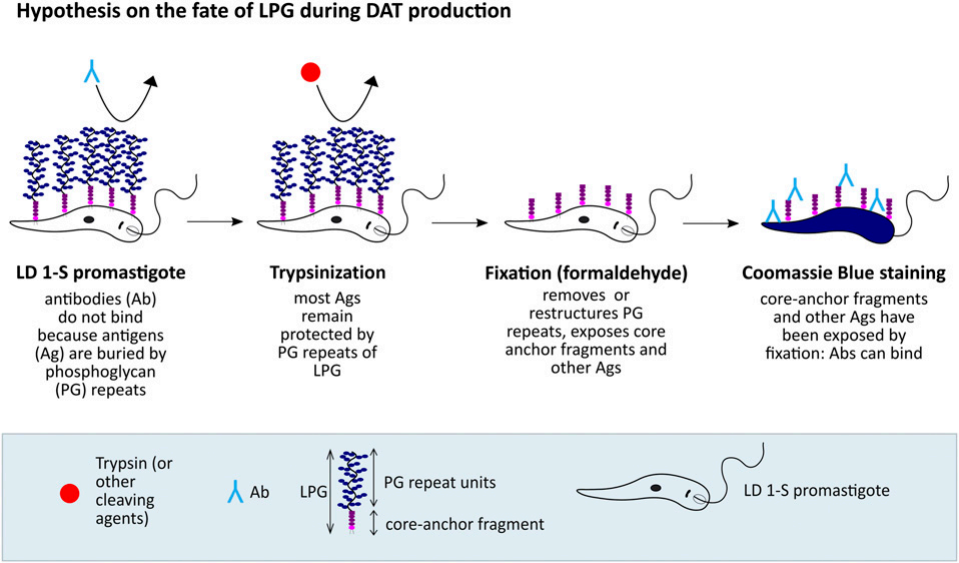
Schematic drawing of the hypothesis on what happens to LPG during the direct agglutination test (DAT) antigen preparation. Ab = antibody; Ag = antigen; LD = *Leishmania donovani*; LPG = lipophosphoglycan; PG = polyglycan. This figure appears in color at www.ajtmh.org.

However, using live LD-1S promastigote microagglutination assays, Karp and others^32^ reported that the LPG-minus mutant R_2_D_2_ agglutinates with higher VL serum dilutions than LPG-bearing parasites from the same original strain. Karp et al.^32^ proposed that LPG masks the antigens buried under the glycocalyx. This was further emphasized by the fact that LPG-containing promastigotes did not stain in immunofluorescence assay with VL sera using live promastigotes, whereas the LPG-minus clone did. Lipophosphoglycan shielding certain molecules (carbohydrates or others) on the promastigotes’ surface is biologically plausible. It has been shown that LPG protects promastigotes against agglutination from sand fly lectins and thus promotes survival in the sand fly host.^33^ Karp et al.^32^ claim that the fixation process during the DAT antigen preparation disrupts this masking effect and exposes promastigote surface antigens recognized by VL serum antibodies; however, they do not provide the data to prove this hypothesis. Importantly, Karp and others showed that they could completely block the reactivity of VL patients’ serum against LPG in ELISA using only the phosphohexasaccharide-lyso-alkyl-phosphatidylinositol (core-anchor) fragment of LPG, as described earlier.^28^ Unfortunately, a similar inhibition experiment has never been performed in DAT. However, the existing results suggest that the major immunogenic region of LPG is the normally unexposed core-anchor region of the molecule.^32^ We may hypothesize that this region is getting exposed during the preparation of the DAT antigen (see schematic drawing Figure 3) and that it is this part that reacts with the monoclonal antibodies against LPG in DAT^20^ and with Mediterranean VL sera in ELISA using purified LPG.^31^ The core-anchor region of LPG is thus a plausible component of the DAT antigen.

### Carbohydrate/glycoprotein.

The core-anchor region of LPG is most likely not the only antigenic component of the DAT as its reported diagnostic accuracy in ELISA does not reach of the reported accuracy of the DAT.^1,31^ However, other carbohydrate components of the glycocalyx might bear epitopes that are specifically recognized by VL sera.

In *T. cruzi*, strong trypsinization removes glycoproteins from the surface.^34,35^ If this was true for *L. donovani* as well, the contribution of carbohydrates to the reactivity of the DAT antigen could be questioned. The complete removal of the glycocalyx by trypsin seems unlikely as no change in ultrastructure of the parasites was seen with transmission electron microscopy when comparing trypsinized and non-trypsinized promastigotes.^36^ More importantly, “no clear differences” were detected when using carbohydrate-binding lectins, such as α-poly-(L)-glutamic acid and concanavalin A (ConA) linked with colloidal gold on trypsinized versus non-trypsinized promastigotes. In 1988, Andrade et al. published two papers on this subject: in a so-called enzyme-linked lectin assay and in an ELISA using peroxidase labeled lectins to compare lectin binding between trypsinized and non-trypsinized promastigotes, they found no differences.^34,37^ Furthermore, a 57-kDa band separated by sodium dodecyl sulfate-polyacrylamide gel electrophoresis (SDS-PAGE) reacted strongly with VL sera and ConA. Trypsin treatment removed the ConA binding but not the antibody binding of this band.^34^

The treatment of the parasites with ME instead of with trypsin before fixation increases the sensitivity and specificity of the DAT.^38^ Mercaptoethanol treatment after trypsinization and fixation has been shown to double the ConA-binding sites of the DAT antigen.^36^ As ME cleaves cysteine bonds, it may cause glycoproteins to unfold and thus expose their carbohydrate moieties and putative epitopes, thus explaining the increased sensitivity of the DAT antigen after ME treatment.

Analysis of *L. donovani* lysates shows the antigenic character of many glycoproteins, for example, gp63 (up to 10% of this surface protein is of carbohydrate nature), the major antigenic band detected by Andrade et al., and a 65-kDa reactive surface antigen detected by Lepay et al.^34,38,39^ El Harith et al.^38^ speculate that the surface glycoprotein (gp63) which is attached to the parasite’s membrane by a GPI anchor is the major DAT antigen. However, when Hommel et al.^20^ used monoclonal antibodies against gp63 in DAT, no agglutination reaction was detected. In ELISA, however, recombinant *Leishmania major* gp63 demonstrates a diagnostic potential for Mediterranean VL with sensitivities around 84–86% and specificities between 97% and 100%.^31,40^ It cannot be excluded that the monoclonal antibodies used by Hommel et al.^19^ were not directed against the major gp63 epitope of the DAT. On the other hand, the recombinant gp63 used by Maalej and others was produced in *E. coli* and, therefore, was not glycosylated. Hence, the performance in ELISA of this recombinant is not due to a carbohydrate epitope.

In summary, carbohydrates are most likely only removed to a small extent by trypsinization. They are exposed by ME treatment, a method that makes the DAT more sensitive. Moreover, many carbohydrates are known *L. donovani* antigens. Taken together, carbohydrates may contribute to the DAT reaction.

### Lipids/hydrophobic molecules.

The Wasserman test uses lipoid extracts from bacteria to detect VL. These lipoid antigens are postulated to detect the nonspecific IgG and IgM raised by tissue damage in infection.^3^ It is possible that the DAT detects similar nonspecific antibodies by exposing lipoid *Leishmania* antigens. As already discussed earlier, the DAT is unlikely to be solely based on nonspecific antibody binding. Yet, these lipid antigens might contribute to the overall agglutination reaction.

Lipids and other hydrophobic molecules constitute a large part of the parasite’s surface. That specific lipids contribute to the DAT agglutination reaction is suggested by a number of observations, although alternative explanations can be formulated. First, the DAT is more sensitive for VL serodiagnosis than a soluble *Leishmania* antigen preparation in ELISA, but this may be due to the different test format.^41,42^ Second, when EL Harith et al.^38^ tried to improve the DAT with different cleaving agents, they noticed a decrease in sensitivity when the antigen was cleaved with triacylglycerol lipase from *Candida cylindracea*. This might, however, be due to lipid-associated molecules being cleaved off, rather than lipids themselves. Slutzky et al.^43^ found the lipid fraction of *L. donovani* to be reactive with human VL sera as compared with healthy human sera, but it may be that the lipid fraction still contained non-lipid antigenic moieties. Overall, the contribution of lipids to the DAT agglutination reaction may be their role in keeping together identical or different antigens on a membrane, thus creating a multiple epitope structure that is destroyed when the membrane is dissolved or disrupted.

### Proteins.

Evidence for the involvement of proteins in the DAT antigen is as ambiguous as for the other classes of compounds. Cleaving agents such as trypsin and pronase do not have a negative effect on the sensitivity of the DAT,^38^ which could be an indication against the importance of protein antigens in the test, as they would be affected by trypsinization. Andrade et al.,^34^ however, suggested that trypsin treatment of *L. donovani* promastigotes did not alter the banding profile in Coomassie Blue–stained SDS-PAGE gels nor did it change immune recognition patterns. Unfortunately, the presentation of the SDS-PAGE gel results renders the interpretation of the data difficult. The possibility that proteolytic agents such as trypsin might not affect proteins, glycoproteins, or other antigens that are shielded by the glycocalyx must be considered. The glycocalyx has been described to protect promastigotes from sand fly lectins, and it might have the same protective effect against other compounds.^33^ This protective effect of the glycocalyx would explain the otherwise puzzling lack of effect on the sensitivity of the DAT antigen by pronase, which cleaves proteins less specifically than trypsin, and by pancreatin, which has a combined proteolytic, lipolytic, and amylolytic effect. One would expect that pancreatin and pronase would cleave off all classes of antigens (proteins, carbohydrates, and lipids) from the promastigote surface and would theoretically leave nothing for antibodies to react with. Yet, the sensitivity of DAT antigen remains unchanged after treatment of the promastigotes with the respective agents.^38^ According to Karp et al.,^32^ the glycocalyx is being removed by the fixation of the promastigotes, which is the step following trypsinization in the DAT preparation process. This removal would make antigens accessible that are otherwise shielded by the glycocalyx (Figure 3). Trypsin has been described to increase the specificity of the DAT antigen by cleaving off nonspecific epitopes from the promastigote surface. This being protein epitopes seems rather unlikely as the use of lipase has the same effect.^38^ Adding to this evidence is the observation that neither pronase nor pancreatin treatment increased the specificity of the DAT antigen.^38^

### Mixture of antigens.

From the available literature, it appears that the DAT antigen is almost certainly composed of multiple *Leishmania*-specific epitopes belonging to a mixture of surface-exposed molecules. This mixture would explain the DAT antigen’s robustness. It is performing well across different geographic regions with different population-related factors and parasite strains as well as across species (*L. donovani* and *L. infantum*), in contrast to the most common single-antigen test for VL, the rK39 test.^1^ The K39 DNA sequence is subject to numerous mutations in different *L. donovani* strains.^44^ Although the rK39 antigen is highly sensitive and specific for VL detection in the Indian subcontinent where genomes of *L. donovani* patient isolates are almost identical,^45^ it loses sensitivity and specificity in regions were *L. donovani* isolates show a high level of heterogeneity, as in Eastern Africa.^1^ In the latter context, a combination of different antigens would create a more universally reactive pool of epitopes and secure sensitivity of the diagnostic test. This is the case in rK28-based immunochromatography, showing promising serodiagnostic accuracy in Eastern Africa.^14–16^ Notably, the DAT has a higher sensitivity than all single antigens known so far.^42^ In their study of agglutination patterns of monoclonal antibodies with the DAT, Hommel et al.^20^ found that the DAT is at least composed of three different antigens: LPG, an HSA*Glut–like epitope, and an additional, not yet described antigen. Strangely enough, DAT makes use of the promastigote form of the parasite, whereas during the course of a human infection with *Leishmania* parasites, promastigotes only persist for a couple of days before transforming into intracellular amastigotes. Therefore, the accuracy of the DAT is presumed to be due to shared antigens between amastigotes and promastigotes. However, LPG from promastigotes is described as an antigenic component of the DAT but is absent in amastigotes. Moreover, when Hommel et al.^20^ immunized rabbits with ex-vivo–purified *L. donovani* amastigotes, the rabbit sera did not react in DAT, although antibody titers were high in ELISA and Indirect Fluorescent Antibody Technique. Sera of rabbits immunized with promastigotes, however, did. This points toward distinct promastigote antigens causing an agglutination reaction in DAT. Whether this is due to patients developing more antibodies during the short promastigote phase, as they are exposed to the immune cells while amastigotes hide in macrophages or to some other reason remains elusive.

## CONCLUSION AND PERSPECTIVES

From what precedes (as summarized in Table 1), it is clear that the identity of the *Leishmania*-specific antigens or epitopes that form the basis of the high diagnostic accuracy of the DAT remains enigmatic. Considering their abundance, it is likely that there are antigenic proteins on the surface of the promastigotes, but there is no clear evidence for or against protein epitopes to be involved in the DAT reaction. Most probably, parts of LPG and potentially other lipid components, as well as carbohydrate epitopes play the major role.

**Table 1:** What is the DAT antigen?

Hypothesis	Pro	Contra	Conclusion
DAT antigen is a non-*Leishmania* antigen			
Nonspecific antigen on promastigote surface or medium contaminants (Wasserman test reinvented)	Wasserman test does not contain *Leishmania* antigens but has high diagnostic accuracy^17^	–	DAT antigen is *Leishmania*-specific but nonspecific antigens might contribute to the reaction
	Molecular mimicry between *Leishmania* antigens and human auto-antigens^24,25^	–	–
	Latex beads coated with glutaraldehyde-cross-linked HSA*Glut agglutinate with VL sera^20^	*Trypanosoma cruzi* epimastigotes prepared in the same way as DAT do not cross-react with VL sera^26^	–
	HAS*Glut in ELISA is more specific for VL than crude promastigote extract^20^	Promastigotes grown in albumin-free medium agglutinate with VL sera and even with a monoclonal antibody against HSA*Glut^22^	–
	Monoclonal antibody against HSA*Glut reacts in DAT^20^	–	–
Nonspecific detection of hyperimmunoglobulinemia (aldehyde test reinvented)	Aldehyde test contains no antigen but has high diagnostic accuracy^18^	–	–
	VL patient serum generally contains high levels of nonspecific immunoglobulins^21^	–	–
	VL patient sera often test false positive in non-VL ELISA^51^	–	–
	DAT reacts with sera from patients with other diseases known to induce hyperimmunoglobulinemia^23^	End-titer of VL sera in DAT is much higher than end-titer of sera from patients with other causes of hyperimmunglobulinemia^23^	–
DAT antigen is an LPG			
	LPG in ELISA has a high diagnostic accuracy to detect VL^31^	LPG masks the antigen on the promastigote surface^32^	Core-anchor fragment of LPG almost certainly DAT antigen
	Monoclonal antibody against LPG reacts in DAT^20^	End-titers of VL sera are higher with LPG-minus mutant *Leishmania* promastigotes than with wild-type promastigotes^32^	–
	LPG reactivity with VL sera in ELISA is completely inhibited by its core-anchor fragment^32^	In immunofluorescence, VL sera react with LPG-minus mutant *Leishmania* promastigotes but not with wild-type promastigotes^32^	–
	Fixation process during DAT preparation disrupts masking effect of LPG^32^ and potentially exposes the LPG core-anchor fragment	–	–
DAT antigen is a carbohydrate or a glycoprotein			
	ME treatment of promastigotes increases ConA-binding sites and DAT sensitivity^36,38^	ME treatment might release carbohydrates as well as other epitopes	Despite trypsin treatment (which removes carbohydrates in *T. cruzi*), carbohydrate involvement in DAT is not excluded, in contrary carbohydrates likely involved in DAT ag
	Trypsin treatment of *Leishmania* promastigotes does not remove the ConA-binding sites of DAT antigen^34,36,37^	–	–
	VL sera react with many *Leishmania* glycoproteins in Western blot^34,38,39^	–	–
gp63 is the DAT antigen	Monoclonal antibodies against gp63 might not be against the epitope in DAT	Monoclonal antibodies against gp63, which has been proposed as DAT epitope,^38^ do not react in DAT ^19^	–
DAT antigen is a lipid			
	Wasserman test uses lipids and has high diagnostic accuracy^17^	–	Lipids are likely involved in the DAT reaction
	Lipase decreases sensitivity of DAT^38^	Other molecules associated to lipids, e.g., embedded in the cell membrane, might be cleaved by lipase as well	–
	DAT is more sensitive than ELISA with hydrosoluble *Leishmania* antigens^41,42^	May be due to differences in test format between ELISA and DAT	–
	Lipid fraction of *Leishmania* parasites is reactive with VL sera^43^	Proteins and carbohydrates remaining in the lipid fraction might react as well	–
DAT antigen is a protein			
	Proteins might be shielded from proteases, such as trypsin, by the glycocalyx, which in turn is removed during fixation of the *Leishmania* promastigotes	Proteases do not have an effect on DAT sensitivity^38^	Protein involvement in DAT reaction is uncertain but not excluded
DAT antigen is a mixture of antigens			
	Geographic diversity of *Leishmania donovani* antigens,^44^ yet high diagnostic accuracy of DAT irrespective of geographic distribution^1^	–	DAT ag is most probably composed of a mixture of antigens
	Studies with monoclonal antibodies show that at least three different epitopes are involved in the DAT test: LPG, an HAS*Glut like epitope, and a yet unidentified epitope^20^	–	–
	“Whole promastigote cells” confer higher sensitivity to DAT than any single antigen known so far^42^	–	–

ConA = concanavalin A; DAT = direct agglutination test; ELISA = enzyme-linked immunosorbent assay; HSA*Glut = human serum albumin; LPG = lipophosphoglycan; ME = mercaptoethanol; VL = visceral leishmaniasis.

Because lipid and carbohydrate molecules most probably play a key role in DAT reactivity, for the development of an RDT with the same diagnostic accuracy as DAT for VL diagnosis in Eastern Africa, classical epitope mapping on purified proteins or in-silico epitope prediction will probably not be successful. Alternative strategies may be more appropriate. One option is to screen random epitope libraries for the so-called mimotopes, molecules that mimic the original epitope. For example, Van Nieuwenhove and coworkers identified diagnostic mimotopes, including one that mimics a conformational epitope of *T. brucei gambiense* VSG, by screening a phage display peptide library with specific antibodies purified from human African trypanosomiasis patients.^46^ Peptide mimotopes have been shown to be able to mimic carbohydrate antigens, for example, by Umair et al.^47^ who used phage display to discover a peptide that mimics a glycan epitope on the surface of parasitic nematode larvae. Recently, by screening a phage display library with antibodies from VL patients, Salles et al.^48^ identified phages and showed their high diagnostic accuracy in ELISA with sera from VL patients and healthy controls from *L. infantum*–endemic regions. Following a similar approach but with antibodies from VL patients’ sera that specifically bind to the DAT antigen(s), one could potentially identify phages that mimic the DAT epitope(s), irrespective of their carbohydrate, lipid, or protein nature.

Alternatives to phage display like the one-bead-one-compound approach have been also used to identify mimotopes, for example, by Leung et al.^49^ to identify mimotopes of the major shrimp allergen tropomyosin. Successful identification of potential carbohydrate antigens has also been demonstrated for *Salmonella enterica* serovars using a pathogen-specific carbohydrate array.^50^

We conclude that even without knowing the exact nature of the DAT antigen(s), it should be possible to develop a novel RDT for VL diagnosis that, particularly in Eastern Africa, is more accurate than those that already are commercially available.
